# bioWeb3D: an online webGL 3D data visualisation tool

**DOI:** 10.1186/1471-2105-14-185

**Published:** 2013-06-07

**Authors:** Jean-Baptiste Pettit, John C Marioni

**Affiliations:** 1 EMBL-EBI, European Molecular Biology Laboratory - European Bioinformatics Institute, CB10 1SD, Cambridge, UK

## Abstract

**Background:**

Data visualization is critical for interpreting biological data. However, in practice it can prove to be a bottleneck for non trained researchers; this is especially true for three dimensional (3D) data representation. Whilst existing software can provide all necessary functionalities to represent and manipulate biological 3D datasets, very few are easily accessible (browser based), cross platform and accessible to non-expert users.

**Results:**

An online HTML5/WebGL based 3D visualisation tool has been developed to allow biologists to quickly and easily view interactive and customizable three dimensional representations of their data along with multiple layers of information. Using the WebGL library Three.js written in Javascript, bioWeb3D allows the simultaneous visualisation of multiple large datasets inputted via a simple JSON, XML or CSV file, which can be read and analysed locally thanks to HTML5 capabilities.

**Conclusions:**

Using basic 3D representation techniques in a technologically innovative context, we provide a program that is not intended to compete with professional 3D representation software, but that instead enables a quick and intuitive representation of reasonably large 3D datasets.

## Background

Visualisation is a key challenge in the analysis of large biological datasets, especially when analysing organized structures with distinct sub-clusters [1]. This is particularly important when analysing 3-Dimensional (3D) datasets. When a biological process or feature has been described spatially by a set of 3D referenced points, either via laboratory work (confocal microscopy for example) or generated within a simulation, with some data attached to each point in space, the first step in interpreting the data is to visualise it. Once the data are visualised and their quality assessed, downstream analysis can proceed. For example, a typical second step is to cluster the observations into different classes based upon the information associated with each point; those results will also need visualisation.

While various 3D visualisation tools have been developed, they have typically been made available via a locally installed piece of software such as BioLayout Express ^3*D*^[2], Arena3D [3], 3D Genome Tuner [4], Amira 3D [5], V3D [6], the Allen Brain Atlas [7] or Cytoscape [8]. These tools are very complete and usually complex to operate for non-expert users. Moreover, they require installation on every machine they are used on, which makes sharing inconvenient. To address this issue, some 3D visualisation tools have been built online and are accessible through the browser directly, such as AstexViewer [9], which is utilised by the Protein Databank Europe via a Java Applet. More recently, visualisation tools developed using HTML5/WebGL capabilities have been described, although they have focused on very specific applications, such as analysing radiology data [10].

Importantly, as yet no tool has allowed biologists to view their own 3D data directly online in an easy, fast, interactive and secure way. Using WebGL and the JavaScript 3D library Three.js, bioWeb3D aims to be a simple, generic, tool for tackling this problem.

## Implementation

bioWeb3D allows the user to represent any 3D dataset on their browser by defining only two files. The two files can either be formatted as JSON or XML files, two widely used structured formats on the web [11,12], or directly as Comma Separated Values files (CSV).

The first file used by the application, referred to as the “dataset file”, contains the coordinates of every point in the dataset. The second type of file used, the “information layer” file, describes one or several information layers that are associated with the points defined in the first file. For example, if each point defines the location of a cell within a tissue, the second file could describe whether a particular gene is expressed in each cell. That way the tissue expression profile can be represented in the spatial context of the tissue.

Datasets can be viewed and compared in up to four “worlds” (each world refers to a separate visualisation sub-window) at the same time. Although browser based, the application, fully written in Javascript, does not need to send any data to the host server. Instead, the modern internet browser’s local file system reading capabilities are used through the HTML 5 FileReader functionality. This allows the application to handle, in a very short period of time, large datasets while ensuring that the privacy of the data is maintained.Although the focus is on making bioWeb3D simple and easy to use, some options are available to customise how datasets are represented. The application can be used to visualise sequential information, such as 3D protein structures, in which case a solid line can be drawn between the points. In other situations, such as when a population of cells is considered, the points are viewed as individual particles. The information layers are visualised by colouring the 3D points according to the class that each point belongs to.

### Technological overview

bioWeb3D is fully written in HTML/Javascript. It relies heavily upon a relatively recent 3D javascript library called Three.js [13]. This library is used as the main interface between WebGL (cross-platform, royalty-free web standard for a low-level 3D graphics API) [14] and javascript. More specifically, bioWeb3D allows the generation and manipulation of simple Three.js objects. Indeed the primary challenge associated with the creation of bioWeb3D has been to design interactions between the 3D visualisation and the user interface in the most efficient way.The 3D data are rendered using simple 2D quadrilaterals positioned in the 3D space according to their coordinates. This simple technique has been selected to keep bioWeb3D as light-weight as possible whilst ensuring good quality visualisation performance and fluidity.

Listing 1 Json dataset file

### Defining the input file formats

JSON is the recommended format to input files into bioWeb3D because of its rigorous structure and its fast object generation, which is directly built into all of the primary internet browsers’ interpreter. Compared to other data-interchange languages, such as XML, JSON is also easily human readable thanks to a light-weight syntax. However, some applications might output data only in an XML format and not JSON, as the latter is generally more web oriented. For this reason bioWeb3D can also accept XML as an input format.Furthermore, much data generated in the biological sciences is stored within CSV files. Converting CSV documents to the JSON or XML format is not always trivial. In order to facilitate this process, the application is also able to directly render simple CSV files that follow a certain format as an input.

#### Dataset file specification

When the user adds a new *Dataset* file, a new Dataset section is created in the “Data” panel of the application. Each dataset file contains one dataset.

##### JSON format

The *dataset* file should have a root object called “dataset” which contains: 

•The “name” property of the dataset (*e.g.*, “my dataset”);

•The “chain” parameter, which should be set to *true* if the points are connected (the default value is *false*) - the data will be considered sequentially, with each point connected by a solid line to the previous and next point according to its order in the dataset file;

•The “points” property, which is a two dimensional array representing a list of (x,y,z) vectors that define the co-ordinates of the points.

Listing 1 is an example of a minimal 3 points dataset file.

Listing 2 XML dataset file

##### XML format

The *dataset* XML format used is very similar to the previously defined JSON format. The file must have a root object called “<dataset>” which contains:

•The “<name>” property of the dataset (*e.g.*, “my dataset”);

•The “<chain>” parameter, which should be set to *true* if the points are linked (the default value is *false*) - the data will be considered sequentially, with each point connected by a solid line to the previous and next point according to its order in the dataset file;

•The “<points>” property, which contains all the single “<point>” elements that define the dataset. Each “<point>” has three properties to define its spatial location, namely “<x>”, “<y>” and “<z>”.

Listing 2 contains the same minimal dataset as Listing 1 but formatted in XML.

##### CSV format

Each line represents a point and the three coordinates on each line must be separated by “comma” characters.

As an example, Listing 3 carries the same information as the JSON file in Listing 1. We note that although the spatial information remains the same it is not possible to set a name or to connect the points within a CSV file input.

Listing 3 CSV dataset file

#### Information layer file specification

The *Information layer* file contains information about the points described in the Dataset file. The information in this file has to be given in the same order as the points defined in the Dataset file.

##### JSON format

The *information layer* files must have a root element named “information”. Since one information file can define multiple information sets, the structure below “information” is a list. Each element of the list is structured as follows: 

•The “name” property (optional);

•The “numClass” property, which indicates the number of different classes the data will be assigned to;

•The “labels” property, which defines a list of names for the “numClass” classes previously defined (optional);

•The “values” property, which defines the class of each point in the dataset. As points do not have single IDs, this property must be in the same order and have the same length as the points defined in the *dataset* file.

For example coming back to the 3 points defined in Listing 1, two information layers could correspond to: 

•one clustering algorithm that puts the first two points together in class one and the third point alone in a second class

•a second clustering algorithm that puts each point in a separate class

Listing 4 JSON information layer file

In this case the Information layer file would look like Listing 4.

##### XML format

The *information layer* XML format used is very similar to the previously defined JSON format. The *information layer* files must have a root element named “<information>”. Since one information file can define multiple information sets, the structure below “<information>” is a list of “<set>” elements. Each “<set>” element is structured as follows: 

•The “<name>” property (optional);

•The “<numClass>” property, which indicates the number of different classes the data will be assigned to;

•The “<labels>” property, which contains as many individual “<label>” properties as the number of different classes. Each “<label>” defines the names for one class (optional);

•The “<values>” property, which contains all the single “<value>” properties, each one defining the class of each point in the dataset. As points do not have single IDs, the “<value>” properties must be in the same order and have the same length as the points defined in the *dataset* file.

Listing 5 XML information layer file

Listing 6 CSV informationlayer file

Listing 5 carries the exact same information as Listing 4.

##### CSV format

Each column represents the class to which a point belongs. The separation character between columns must be a “comma”. Listing 6 carries the same information as Listing 4. Note that it is not possible to use the “labels” or “name” properties available in Listing 4 within a CSV information layer file.

## Results and discussion

### Basic usage

**Figure 1:**
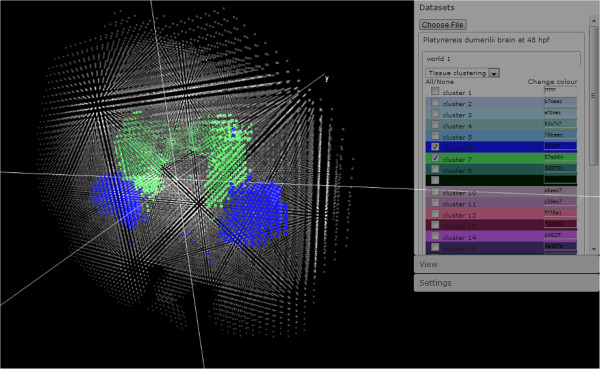
**An example of the application of bioWeb3D.** The 3D location of cells within the brain of the marine annelid *Platynereis dumerilii* is shown. Two classes are displayed (in green and blue) along with the shadow of the remaining cells. The User interface is visible on the right of the screen and can be hidden. Data for this figure was taken from [15].

The goal of bioWeb3D is to allow scientists unfamiliar with visualisation software to explore 3D data very quickly without having to install any software. To illustrate its utility we applied bioWeb3D to study heterogeneity in gene expression levels across cells in the brain of the marine annelid *Platynereis dumerilii*. Using a newly developed technique called PrImR [15], Tomer and colleagues were able to generate a map of pseudo-cells within the *Platynereis dumerilii* brain, before determining whether a pre-defined set of genes were expressed in each pseudo-cell. In the context of bioWeb3D, the locations of the pseudo-cells are used to generate the “Dataset” file and information about the sets of cells that define clusters with similar gene expression profiles are used to generate the “Information Layer” file. In Figure 1 we illustrate the results — each point represents a pseudo-cell and its colour indicates the class (or cluster) to which it belongs to.

bioWeb3D can be used to visualise datasets derived from a wide variety of biological assays. Examples are shown on the Github wiki [16], where we display a 3D representation of a Principal Component Analysis (PCA) carried out with R and the 3D structure of a protein extracted from the PDBe database.

More generally, the user can interact with the visualisation via an interface on the right of the screen, which contains three panels. In the “dataset” panel, the user can choose the *datasets* and *information layer* files that should be represented in each world. This panel also allows the user to show/hide specific classes of the selected information layers. Each dataset file entered will create a new sub-panel where the user can input *information layer* files for that world. Selecting an *information layer* in the drop-down list will display the data in the current world and generate a list of classes that the user can modify regarding their visibility and colour. The “View” panel enables the user to choose which of the worlds are shown on the screen, ranging from 1 to 4. Finally, the “Settings” panel provides the user with a number of options that affect all worlds and all datasets, such as modifying the axes scales, modifiying the transparency and size of raw data points and information layer coloured points. The user can also choose to enable centering of the data around 0 or leave the coordinates as inputted.

### bioWeb3D and local software

Many 3D visualisation software tools, most of which require local installation, exist and provide similar functionalities with standard 3D format input such as Wavefront.OBJ. Some are extremely generic and powerful like Blender or Amira 3D. However, these tools are not typically oriented towards a scientific audience. Moreover, those that are more focused on science are often targeted towards a very specific application, especially in the medical sciences [4]. In this context, we believe that bioWeb3D can be useful as it is completely generic and browser based. It should also be noted that recent browser improvements regarding GPU acceleration through the WebGL paradigm allow bioWeb3D to visualise several hundred thousand points. Additionally, local software is usually platform specific, which is not the case for browser based applications.

### bioWeb3D and java applets

As mentioned previously, browser based 3D visualisation tools currently exist mainly in the form of Java Applets. This technology has attracted much criticism in 2012 regarding security flaws, leading the “United States Computer Emergency Readiness Team” to advise that all Java Applets should be disabled due to current and future Java vulnerabilities [17]. The development of WebGL technology is viewed by many as a candidate for replacing Applets.

### Current limitations

The main current limitation of a WebGL based application is the machine and browser compatibility. Only computers with fairly recent graphic cards will be able to run a 3D environment. It should also be noted that Microsoft has notified the developer community that Internet Explorer is not scheduled to support WebGL in the near future. However, importantly, Chrome, Firefox, Safari and Opera all now support WebGL applications. Moreover, we note that WebGL is also supported on mobile platforms such as iOS or Android [18].

### Open source and collaborative development

As a fully open source software, the source code for bioWeb3D is available on Github [16], a web platform that allows interested parties to collaborate on the development of the project. In the wiki page “Contribute to bioWeb3D”, directions to alter or add capabilities to bioWeb3D are provided for users who wish to get involved.

## Conclusions

bioWeb3D is designed to be a simple and quick way to view 3D data with a specific focus on biological applications. Being browser-based, the software can be easily used from any computer without the need to install a piece of software. Importantly, bioWeb3D has been designed to offer a very straightforward and easy-to-use working environment. Despite current limitations in terms of compatibility or rendering performance for large numbers of points, we believe that bioWeb3D will enable non-experts in 3D data representation to quickly visualise their data and the information attached to it in many biological contexts, thus facilitating downstream analyses.

## Availability and requirements

The full source code is available on the Github page of the project [16]. A live version of the software is online [19]. You will require a graphical card and a browser with WebGL capabilities to run bioWeb3D.

**Project name:** bioWeb3D

**Project home page:**http://www.ebi.ac.uk/~jbpettit/bioWeb3D

**Operating system:** Platform independent

**Programming language:** HTLM/Javascript

**Other requirements:** Browser and graphic card with WebGL capabilities

**License:** Academic Free License ("AFL") v. 3.0

## Competing interests

The authors declare no competing interests.

## Authors’ contributions

JBP developed the presented software and drafted the manuscript. JCM has been involved in writing and editing the manuscript. He has also given the final approval of this version to be submitted. All authors read and approved the final manuscript.
